# Screening of colistin-resistant bacteria in livestock animals from France

**DOI:** 10.1186/s13567-022-01113-1

**Published:** 2022-11-22

**Authors:** Afaf Hamame, Bernard Davoust, Bouthaina Hasnaoui, David Lupande Mwenebitu, Jean-Marc Rolain, Seydina M. Diene

**Affiliations:** 1 APHM, MEPHI, Faculté de Pharmacie, Aix Marseille Université, IRD, 19-21 Boulevard Jean Moulin, 13005 Marseille, France; 2 IHU-Méditerranée Infection, 19-21 Boulevard Jean Moulin, 13005 Marseille, France; 3 VITROME, 19-21 Boulevard Jean Moulin, 13005 Marseille, France

**Keywords:** colistin-resistant bacteria, *mcr* genes, animals, France

## Abstract

Colistin is frequently used as a growth factor or treatment against infectious bacterial diseases in animals. The Veterinary Division of the European Medicines Agency (EMA) restricted colistin use as a second-line treatment to reduce colistin resistance. In 2020, 282 faecal samples were collected from chickens, cattle, sheep, goats, and pigs in the south of France. In order to track the emergence of mobilized colistin resistant (*mcr*) genes in pigs, 111 samples were re-collected in 2021 and included pig faeces, food, and water from the same location. All samples were cultured in a selective Lucie Bardet Jean-Marc Rolain (LBJMR) medium and colonies were identified using MALDI-TOF mass spectrometry and then antibiotic susceptibility tests were performed. PCR and Sanger sequencing were performed to screen for the presence of *mcr* genes. The selective culture revealed the presence of 397 bacteria corresponding to 35 different bacterial species including Gram-negative and Gram-positive. Pigs had the highest prevalence of colistin-resistant bacteria with an abundance of intrinsically colistin-resistant bacteria and from these samples one strain harbouring both *mcr-*1 and *mcr-*3 has been isolated. The second collection allowed us to identify 304 bacteria and revealed the spread of *mcr-*1 and *mcr-*3 in pigs. In the other samples, naturally, colistin-resistant bacteria were more frequent, nevertheless the *mcr-*1 variant was the most abundant gene found in chicken, sheep, and goat samples and one cattle sample was positive for the *mcr-*3 gene. Animals are potential reservoir of colistin-resistant bacteria which varies from one animal to another. Interventions and alternative options are required to reduce the emergence of colistin resistance and to avoid zoonotic transmissions.

## Introduction

Colistin (polymyxin E) is a cationic polypeptide antibiotic used as a last-line therapeutic drug, to treat bacterial infections, especially carbapenem-resistant Gram-negative bacteria [1].

Colistin has been used for decades in veterinary medicine as a growth factor [2]. Thus, the high spread of colistin resistance has been related to colistin use via selection pressure in the ecosystem [3]. The European Medicine Agency has suggested limiting colistin use because it plays a crucial role in the exclusion of colistin resistance in epidemiological studies [4].

Colistin resistance is mediated by different mechanisms that include chromosomal mutations, mobile genetic elements (MGEs) harbouring mobilized colistin resistant (*mcr*) genes (transposon, integron, plasmid), efflux pumps, and even vesicles [5]. First of all, colistin resistance was reported to be due to regulatory modification mediated by chromosomal gene mutations (*mgrB*, *pmrAB*, *phoPQ*) [6]. Then, in 2015 Chinese researchers reported the first plasmid-mediated colistin resistance, harbouring the *mcr-*1 gene, which has since propagated to 20 other countries [7]. *mcr* genes have dispersed worldwide in different ecosystems [8, 9]. Since 2015, several variants of mobile colistin resistance gene have been discovered ranging from *mcr-*2 to *mcr-*10 [10–18]. Recently, in 2022, subvariants of *mcr* genes have been discovered by metagenomic analysis [8]. Usually, *mcr* genes are transported by plasmids such as IncI2, IncHI2, IncX4, IncP, IncF, and IncY which have a high potential for transmission [19].

In contrast, a retrospective study found the *mcr-*1 gene in *Escherichia coli* isolated from poultry in the 1980s, when colistin first started to be used in food-producing animals in China. One hypothesis is that this is due to the use of polymyxin E in the animal industry [20]. Several studies have suggested that the transmission of *mcr-*1 in human beings is caused by zoonotic transmission, especially since colistin use in humans was banned [21]. It should be noted that animals are in direct and indirect contact with humans, whether for food consumption or as companionship. The contact between the environment, animals, humans and the eco-system exposes human beings to the zoonotic transmission of antibiotic resistance factors, either bacteria with intrinsic resistance or bacteria with resistance which is acquired via MGEs [22–24].

Over the last decade, colistin resistance in many bacterial species has been widely reported around the world [25]. However, information on the prevalence of bacteria that are resistant to critically important antimicrobials in animals is lacking in France. Recently, Dufreche provided a statistical estimate of veterinary antibiotic consumption in France (422 tons of antibiotics) [26]. The current study aims to screen colistin-resistant bacteria isolated from domestic animals including chickens, cattle, goats, sheep, and pigs. Furthermore, this study is performed in the context of the French antimicrobial resistant strains surveillance network.

## Materials and methods

### Sample collection and ethics authorization statement

**Figure 1:**
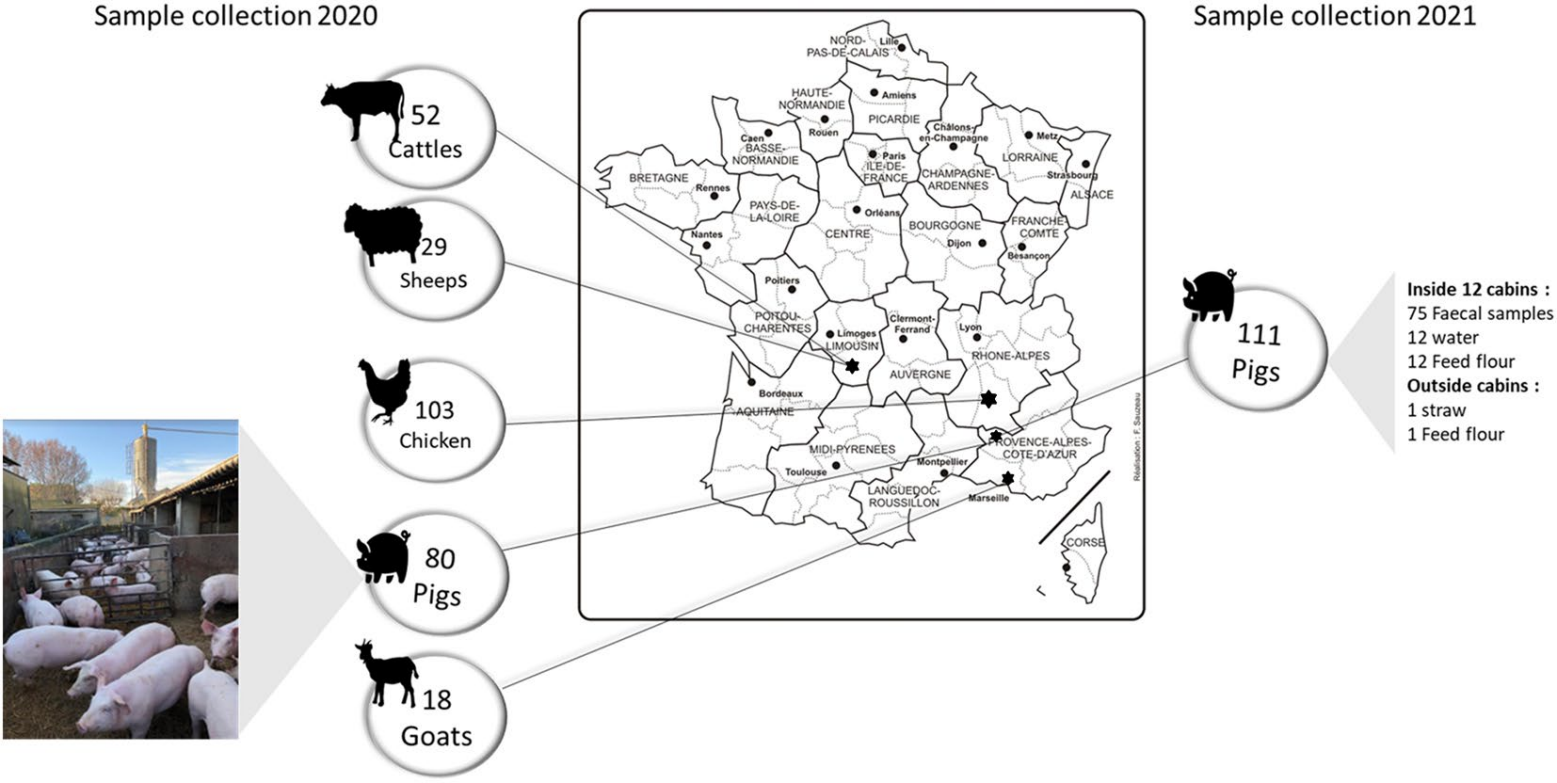
**Geographical map showing the provenance of animal samples.** The numbers in the bubbles represent the number of collected samples. The samples collected in 2020 are faecal samples only, while those collected in 2021 are from different origins. The localizations from where samples were collected are indicated by black stars.

Between 2019 and 2020, faecal samples were collected from four different counties in France. To get fresh stools, the faeces were collected with the assistance of a veterinarian. A sterile cotton swab and a wooden medical spatula were used to collect one faecal sample per animal. The veterinarians reported that animals in France were under standard rules of hygiene, food consumption, and restricted antibiotic use. 282 samples were collected using sterile tubes and sterile spatula from five species of animals: chickens from the Drome county; goats from Bouches-du Rhône; cattle and sheep from the Creuse; pigs from Vaucluse. Samples from goats were pelleted but were crushed with sterile water before analysis. As a part of the epidemiological monitoring of faecal and food samples from pigs, 111 additional pig samples were collected one year after the first collection (i.e., in 2021). All the collected samples were stored at —80 °C for later use. The number of samples taken for each animal is presented in detail in Figure 1.

A Prefectorial authorisation (Bouches-du-Rhône) No.13 205 107 of 4 September 2014 authorises the IHU-Méditerranée Infection to use unprocessed animal by-products in categories 1, 2, and 3 for research and diagnostic purposes. Another order of 8 December 2011 lays down the health rules concerning animal by-products and derived products pursuant to the application of Regulation (EC) No. 1069/2009 and Regulation (EU) No 142/2011.

### Screening and identification of colistin-resistant bacteria

All the samples were suspended in Tryptic Soy Broth medium (TSB) for bacterial enrichment and then cultured on Lucie Bardet Jean-Marc Rolain medium (LBJMR). LBJMR medium contains purple agar supplemented with glucose as a fermentative substrate. This medium was used as a selective medium containing 4 µg/mL of colistin sulphate salt and 50 µg/mL of vancomycin. In the plate agar, the *Enterobacteriaceae* appears yellow, contrasting with the purple agar with a size between 2 and 3 mm. *Enterococci* strains were round and small in size at 0.1–1 mm [27]. The strain set is distinguished according to Gram-negative bacteria (GNB) and Gram-positive bacteria (GPB). GPB are naturally colistin-resistant bacteria, while (GNB) are either naturally resistant to colistin or have acquired resistance via different mechanisms.

Colonies with different morphologies were selected from the selective agar plate. The isolated bacteria were then identified using a Microflex LS spectrometer (Bruker Daltonics, Bremen, Germany). Isolates were efficiently identified when the score values ranged from 2.3 to 3.0. This identification depended on Culturomics, BDAL, and Timone databases. The bacteria with low scores identification due to their fatty texture were subjected to protein extraction in order to improve their score.

### Antimicrobial susceptibility test (AST)

All GNB isolates which were non-naturally colistin-resistant and grown on the LBJMR medium were subjected to an AST according to the current (DD) test method (Kirby-Bauer procedure). The minimal inhibition concentration (MIC) was confirmed by CLSI and EUCAST guidelines [28]. AST was performed with a definite turbidity bacterial suspension in NaCl (0.5 McFarland; 1.5 × 10^8^ cells/mL). Antibiogram test included the following sixteen antibiotics: amoxicillin (AMX), amoxicillin-clavulanic acid (AMC), cefepime (FEP), piperacillin/tazobactam (TPZ), cefalotin (KF), ceftriaxone (CRO), ertapenem (ETP), imipenem (IMP), fosfomycin (FF), nitrofurantoin (F), trimethoprim-sulfamethoxazole (SXT), amikacin (AK), ciprofloxacin (CIP), doxycycline (DO), colistin (CT), and gentamicin (GT) (Bio-Rad, Marne-la-Coquette, France). Hierarchical clustering of the antibiotic resistance phenotype was performed using Multi-Experiment Viewer (MeV 4.9.0).

Strains with a narrow diameter zone of inhibition (ZOI) less than 15 mm were picked out to confirm the minimal inhibition concentration value using other complementary tests, namely the E-tests method (BioMérieux) and UMIC test (Biocentric Bandol, France) [29]. Furthermore, strains were considered to be multidrug-resistant (MDR) if bacteria were resistant to more than three different classes of antibiotics.

### Screening of colistin resistance genes

All bacteria with colistin MIC_col_ ≥ 2 μg/mL as well as naturally resistant bacteria were subjected to several bio-molecular tests to screen for the following *mcr* genes: *mcr-*1, *mcr-*2*, mcr-*3, *mcr-*4, *mcr-*5 and *mcr-*8 [30]. It should be noted that naturally colistin-resistant bacteria can carry *mcr* genes such as *Proteus mirabilis* [31].

Bacterial DNA was first extracted using the EZ1 DNeasy Blood Tissue Kit (Qiagen GmbH, Hilden, Germany) [32]. The absorbance measurements for DNA purity ranged from 260 to 280 nm (Spectrophotometer ND-100, Nanodrop Thermo Fisher Scientific, Wilmington, DE, USA). The *mcr* genes were then detected using Real-Time Reaction. qPCR using CFX96 TM Real-time system/C. A positive control template was included in each qPCR with *E. coli* and *Klebsiella pneumoniae* carrying *mcr* genes as a positive control and *E. coli* ATCC 25 922 for the negative control. Strains were considered positive when the cycle threshold value of real-time PCR was ≤ 30. qPCR results were confirmed by ST-PCR and Sanger sequencing with blast and alignment analysis of the *mcr* genes sequence with ≥ 90% identity.

### Genomic sequencing and bioinformatic analysis

The whole-genome sequencing of interest was performed using next-generation sequencing tools (NGS). The Illumina MiSeq sequencer (Illumina, San Diego, CA, USA) and Oxford Nanopore GridION sequencing were performed to have ultra-deep and best-quality reads [33, 34]. The sequenced genomes were assembled using Spades 3.5.0 software [35] and genome annotation was performed using Prokka [36]. Antibiotic resistance genes were investigated using different databases, including Resfinder [37], ARG-ANNOT [38], Card [39], and Plasmid Finder [40].

### Descriptive and comparative statistical analysis

All the colistin-resistant bacteria were devised into two populations according to the Gram (GNB/GPB). The GNB colistin resistance population segregated into naturally and acquired colistin-resistant bacteria by two different mechanisms. Each criterion is represented by a number value of bacterial species. The result values were expressed as relative frequency (percentage) in each relevant animal population.

## Results

### Screening of colistin-resistant bacteria in animals

Culture on LBJRM selective medium allows isolation of a wide variety of colistin-resistant bacterial species in domestic animals. The results of the first round of samples collections between 2019 and 2020 from chicken, cattle, goat, sheep, and pigs yielded to 397 bacterial isolates composed by 35 different bacterial species from the LBJMR agar plates.

In the current study, for the chicken samples, 96% (*n* = 109) of 113 isolated strains were GNB. The dominant strains were naturally colistin-resistant: 75% of GNB were *P. mirabilis* and *P. vulgaris*. The GNB with acquired colistin resistance in chicken samples were: 12% (*n* = 13) *E. coli*, 4% (*n* = 5) *Pseudomonas fragi*, 4% (*n* = 5) *P. lundensis*, 2% (*n* = 2) *Ewingella americana* and 1% (*n* = 1) *Citrobacter freundii*.

Concerning faecal samples from cattle, 101 colistin-resistant bacteria were isolated and 73% of the isolates were GNB and 27% were GPB. 66% of GNB were naturally colistin-resistant bacteria including 7 *Hafnia alvei* and 42 *P. vulgaris*. GNB with acquired colistin resistance were 1% (*n* = 1) *Achromobacter insolitus*, 1% (*n* = 1) *C. braakii*, 1% (*n* = 1) *C. freundii*, 1% (*n* = 1) *Enterobacter cloacae*, 20% (*n* = 15) *E. coli*, 4% (*n* = 3) *P. putida* and 4% (*n* = 3) *Yersinia entercolitica.*

37 colistin-resistant bacteria were isolated and identified from goat samples. Of these, 95% were GNB and the naturally colistin-resistant strains were 3% (*n* = 1) *Brucella grignonense* and 60% (*n* = 21) *H. alvei*. In contrast, acquired colistin resistance in these GNB concerned 28% (*n* = 10) *E. coli*, 3% (*n* = 1) *P. abietaniphila* and 6% (*n* = 2) *P. putida*.

From sheep samples, 13 colistin-resistant bacteria were isolated, including 11 *E. coli*, 1 *B. grignonense*, and 1 *H. alvei*.

**Figure 2:**
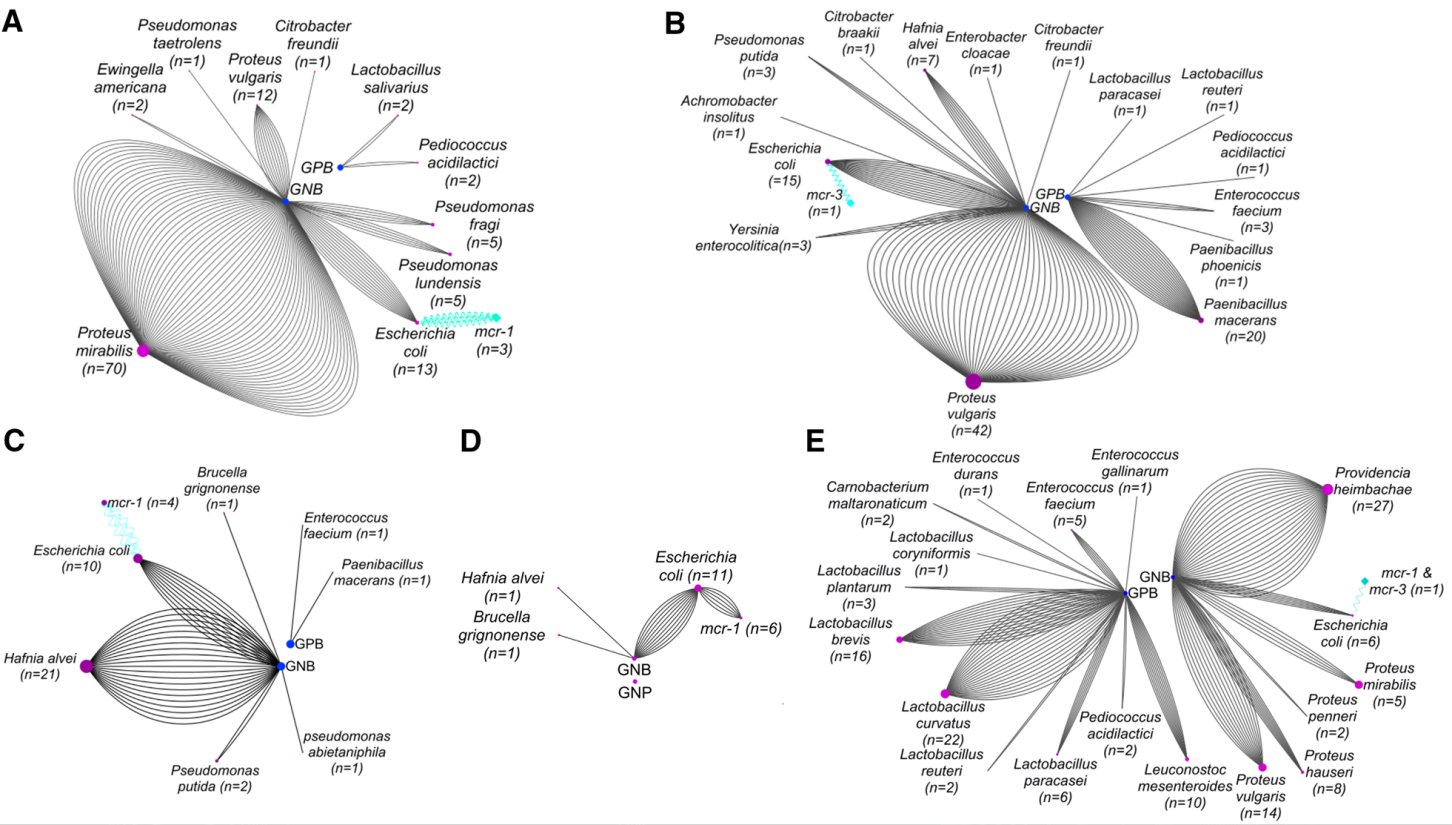
**Network screening analysis of colistin-resistant bacteria isolated from faecal samples of domestic animals in France using Cytoscape 3.9.0.** **A** Isolated colistin-resistant bacteria from chicken. **B** From cattle; **C** From goats; **D** From sheep; **E** From pigs. Colistin-resistant bacteria are divided into two batches according to the Gram GNB and GPB. Bacteria carrying *mcr* genes are distinguished by blue zigzag arrows (edge). The number of edges for each bacterial species represents the number of isolated bacteria. The size of nodes also shows the variable number of isolated bacteria.

Regarding pigs, 53% (*n* = 71) of 133 isolated bacteria were GPB and 47% (*n* = 62) were GNB. 90% of GNB which were naturally colistin-resistant were 43% (*n* = 27) *Providencia heimbachae*, 47% (*n* = 29) *P. vulgaris*, *P. hauseri*, *P. mirabilis* and *P. penneri.* For acquired colistin resistance only 6 *E. coli* were isolated. The epidemiological results of identified bacteria in animals are illustrated in Figure 2.

**Figure 3:**
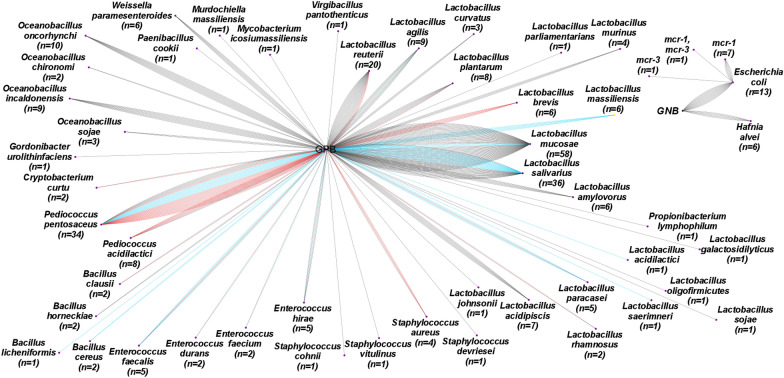
**Screening of colistin-resistant bacteria isolated from collected samples of pigs in 2021.** Edges in black represent faeces samples, red for food, and blue for water. Bacteria are devised into two groups according to the Gram, *mcr* genes cross-link with *E. coli.*

Indeed, 304 colistin-resistant bacteria were isolated from the second collection of pig samples conducted in 2021. Of the isolated bacteria, 94% were GPB. 60% (*n* = 176) of GPB were species of the genus *Lactobacillus*, found in the three types of samples (food, water, and stools). It should be noted that the probiotics used as a growth factor contained biomass of *Lactobacillus* which explains the propagation of this bacterial genus in pigs. A double cross-link of bacteria between food and faeces was found in the following species *L. reuterii*, *L. plantarum*, *L. rahmnosus*, *Staphylococcus aureus*, *Bacillus clausii*, *Enterococcus faecalis*, *Pediococcus pentosaceus*, *P. acidilactici*, and *Cryptobacterium curtu.* In addition, the cross-link between water and faeces was detected in *L. agilis*, *L. mucosae*, *L. salivarius*, *E. hirae*, *E. faecalis*, and *P. pentosaceus.* In contrast, a triple cross-link of bacteria was observed in *P. pentosaceus.* In one faecal sample, one *Mycobacterium icosiumassiliensis* (*n* = 1) was identified. Regarding, GNB 13 *E. coli* and 6 *H. alvei* were identified. Figure 3 illustrates the screening of colistin-resistant bacteria in pigs (faeces, feed, and water).

### Phenotype of antibiotic resistance

The isolated bacteria with acquired colistin resistance were tested with E-test. All the tested bacteria had a minimal inhibition concentration MIC ≥ 2 and were therefore resistant to colistin. A series of antibiogram tests were carried out to determine the most common antibiotic resistance phenotype in colistin-resistant bacteria in animals.

All the isolated bacteria from the LBJMR medium were confirmed to be resistant to colistin with an inhibition zone diameter (ZOI) ≤ 15 mm. 86% and 64% of tested bacteria were resistant to amoxicillin and amoxicillin-clavulanic acid respectively, and included *E. coli*, *P. lundensis*, *P. heimbachae*, *P. putida*, *P. fragi*, *A. insolitus*, *C. brakii* and *Y. enterocolitica.* 34% of the isolates were resistant to piperacillin/tazobactam and included 1 *C. freundii*, 1 *E. cloacae*, 1 *E. coli*, 2 *E. americana*, 13 *P. heimbachae*, 5 *P. fragi*, 1 *P. lundensis* and 1 *P. putida.*

**Figure 4:**
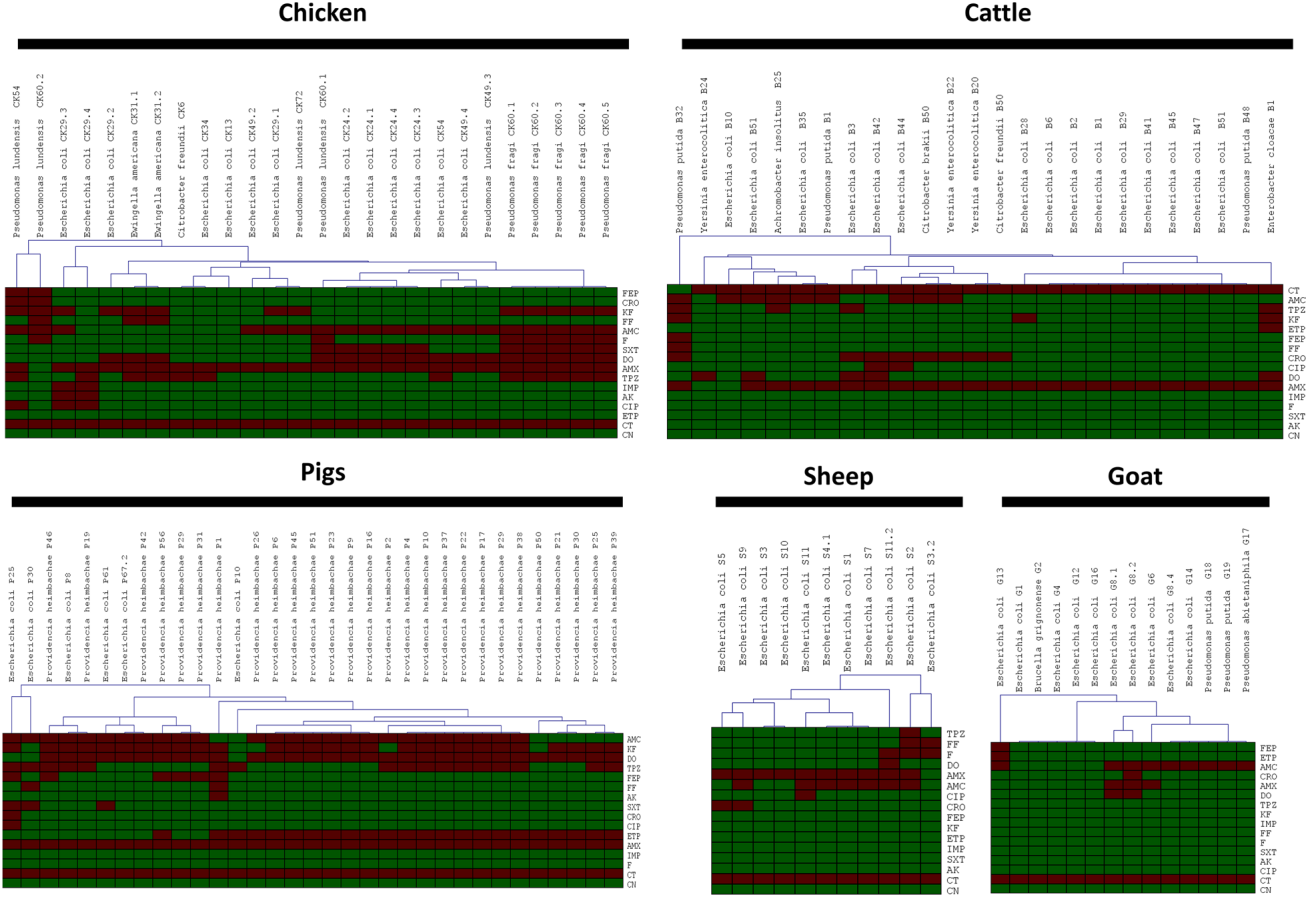
**Hierarchical clustering analysis of antibiotic resistance phenotype of colistin-resistant bacteria isolated from domestic animals in France using MEV 4.9.0 software.** The green colour refers to the sensitive phenotype of the bacteria to the antibiotic, and the red colour refer to resistance. amoxicillin (AMX), amoxicillin-clavulanic acid (AMC), cefepime (FEP), piperacillin/tazobactam (TPZ), cefalotin (KF), ceftriaxone (CRO), ertapenem (ETP), imipenem (IMP), fosfomycin (FF), nitrofurantoin (F), trimethoprim-sulfamethoxazole (SXT), amikacin (AK), ciprofloxacin (CIP), doxycycline (DO), colistin (CT), and gentamicin (GN).

Regarding the cephalosporin family, 8% of bacteria were resistant to cefepime including 1 *E. coli*, 5 *P. heimbachae*, 1 *P. putida* and 2 *P. lundensis.* 17% were resistant to cefalotin and 12% were resistant to ceftriaxone. Interestingly, resistance to carbapenems was observed in this study. Indeed, 23% of bacteria were resistant to ertapenem including 22 *P. heimbachae*, 2 *E. coli*, and 1 *E. cloacae.* According to this study, 5% of the strains were resistant to fosfomycin and nitrofurantoin, including 3 *E. coli*, 1 *P. putida*, 1 *P. lundensis* and 1 *P. heimbachae.* Furthermore, less than 34% of tested bacteria were resistant to ciprofloxacin, amikacin, doxycycline, and gentamicin. The results of all antibiotic resistance phenotype are presented in Figure 4.

### Screening of colistin resistance *mcr* genes

In this study, *mcr-*1 was detected in almost all animal samples. The detection of *mcr* genes was confirmed by three polyphasic approaches. The CT values of the RT-PCR assays which were performed on all bacteria carrying *mcr* genes were less than 30 and all the screening results were confirmed by standard-PCR, by Sanger sequencing, and sequence analysis that revealed % of nucleotide identity ≥ 90 with the different *mcr* gene variants.

**Figure 5:**
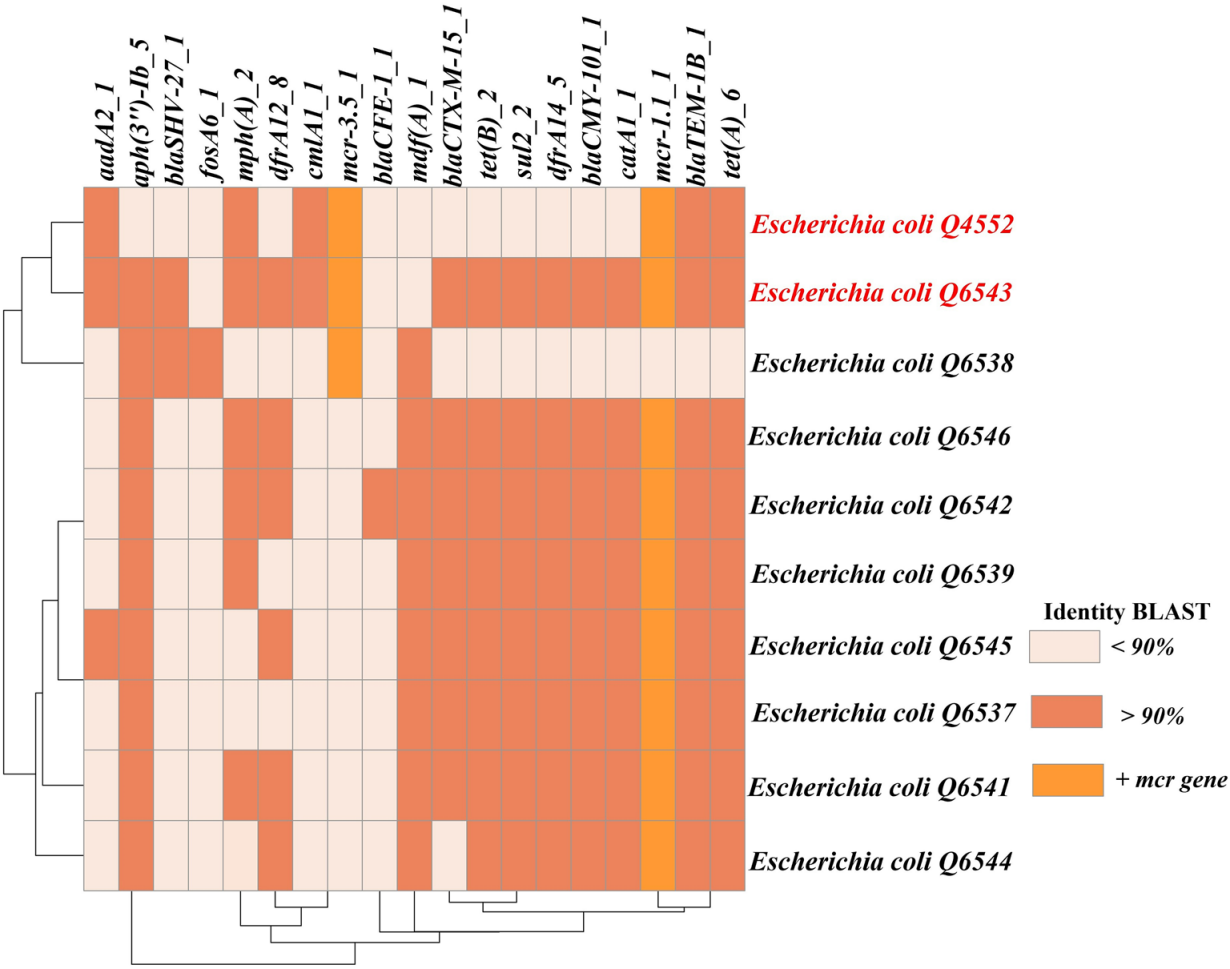
**Annotation heatmap of antibiotic resistance genes found in** ***E. coli* harbouring* mcr* genes isolated from faeces of pigs.**

In faecal samples from chickens, the *mcr*-1 gene was detected in three *E. coli* isolates. Regarding cattle, the *mcr-*3 gene was found in three *E. coli* isolates*.* For the goat samples, the *mcr-*1 gene was detected in four *E. coli* isolates, while in the sheep samples, this gene was identified from 6 *E. coli* isolates. In the pig samples, an atypical *n* = 1 *E. coli* harbouring two *mcr* variants both *mcr-*1.1 and *mcr-*3.5 was isolated and we recently described its genomic characterisation [41]_._ In contrast, one year after the *mcr* genes were disseminated alongside the pig samples, nine *E. coli* were isolated, harbouring different variants of the *mcr* gene, including 7 *mcr-*1, 1 *mcr-*3, and the co-presence of one *mcr-*1*/mcr-*3. Those strains were MDR isolates with various antibiotic resistance genes against more than three antibiotic families (Figure 5). Furthermore, overall isolates carrying *mcr* genes have (MIC ≥ 4 mg/L) and qPCR CT values less than 30.

## Discussion

In this study, *E. coli* strains harbouring colistin resistance *mcr* genes were abundant in all animals. Thus, *E. coli* is one of the pathogenic bacteria agents in animals, particularly farm animals [42]. All *E. coli* strains can induce and cause nosocomial diseases associated with symptoms such as neonatal diarrhoea, post-weaning diarrhoea (PWD), and other pathologies including disease (OD), septicaemia, polyserositis, mastitis, and urinary tract infections [43]. In France, colistin-resistant *E. coli* from diseased pigs harbouring mcr**-**1 have been already reported [44]. Furthermore, colistin resistance in *Salmonella* spp is frequently low in comparison to the proportion of intrinsically colistin-resistant bacteria that were isolated from healthy animals including pigs, cattle, and poultry in different countries [2]. A previous study reported the dominance of GNB colistin-resistant strains in western France [45]. Since the discovery of the first plasmid carrying the *mcr-*1 gene in pigs from China, colistin resistance genes disseminated over the world, and animal gut became a source of colistin resistance [7]. Moreover, the discovery of the *mcr-*2 gene in Belgium was followed by the dissemination of colistin resistance across Europe [10]. However, despite the increasing number of studies describing mobile colistin-resistance genes, the relative frequency of natural resistance is found to be higher than acquired resistance. The faecal carriage of GNB with acquired colistin resistance was low with 1.4% in the presence of high intrinsically colistin-resistant bacteria with 23% [45]. One of the most recent studies of colistin resistance genes in pigs took place in 2009 and 2013, in which the *mcr-*1 gene was detected in 70 out of the 79 investigated pig samples [4]. Indeed *mcr-*1 and *mcr*-3 were found in *E. coli* also carrying *bla*_CTX-M-55_, which were isolated from healthy French cows in the IncF18 and IncF46 plasmids, respectively [46]. Between 2005 and 2014, the co-occurrence of the *mcr-*1 gene and extended-spectrum-β-lactamases (ESBL) from faeces of diarrhoeic veal calves was reported in France with a potential zoonotic transmission [47]. Furthermore, the emergence of colistin resistance worldwide is not necessarily related to colistin use. The transmission of colistin resistance is usually due to the zoonotic transmission of colistin-resistant genes via MGEs from animals to humans [48]. However, colistin selection pressure had a major role in colistin resistance due to the long use of this antibiotic as a growth promoter and as an antibiotic against carbapenem-resistant bacteria, causing various infectious diseases [2]. Sometimes, the recombination of several antibiotics is necessary when it concerns MDR bacteria such as *K. pneumoniae* reported in France carrying the *mcr* gene, *bla*_OXA-48,_ and *bla*_CTX-M-15_ [49]. Many reviews have summarised the status of colistin resistance in animals around the world. In over 30 countries across five continents, the most prevalent colistin resistance genes are principally *mcr-*1 in western and southern Europe (Spain, Portugal, Germany, and Italy) [50]. In 2013, European countries estimated that the percentage of resistance to colistin in *E. coli* strains isolated from the digestive tract microbiota of healthy animals remained < 1% [51]. *Salmonella* spp and *E. coli* isolated from poultry in Italy have a sporadic instance of high colistin resistance levels [52]. The colistin resistance in animal food from Denmark is low due to the strict colistin use in their farms [53]. In Italy, turkeys had a greater prevalence of *mcr-*1 in *E. coli* (21.9%) compared to broilers (2%) and layer hens (9%) [52]. Between 2012 and 2016, a triple co-occurrence of *mcr* genes has been reported in healthy pigs, cattle, and poultry faeces in Belgium [54]. *E. coli* isolated from pigs and white stork in Spain has been reported as an *mcr* carriers in IncX4, IncHI2, and IncI2 [55].

Since 2012, animals have also been a discreet reservoir of colistin resistance in Brazil in food-producing animals (chicken, swine, cattle, goat) and companion animals (cats, dogs, horses) [56]. Remarkably, chickens, which are the principal animals for food consumption, show the greatest emergence of the *mcr* genes [20, 21, 57]. In 2021 in Iran, 607 *E. coli* isolates collected from broilers, ostriches, cattle, sheep, pigeons, and dogs were found to be carriers of plasmid-mediated colistin resistance genes (*mcr-*1 and *mcr-*2) [58]. The zoonotic transmission of *mcr* genes from pets to humans has been widely reported [59]. The transmission of colistin resistance genes between dogs and their owners, containing significant quantities of positive *E. coli* with the co-occurrence of *bla*_CTX-M_ and *mcr* genes, has been detected in China [60]. It should be noted that *mcr* genes in dogs and cats have recently been reported in France [30]. Colistin resistance has also frequently been found in river water and vegetable samples in Switzerland [61] and other studies have found *mcr* genes in water in Malaysia [62]. A top public health goal is preventing the spread of colistin-resistant bacteria through zoonotic transmission.

In the current study, we concretely report the screening of colistin-resistant bacteria in animals. One limitation of this work can be highlighted concerning the use of the selective medium with colistin concentration of 4 μg/mL that may prevent the growth of bacteria with low colistin MIC (less than 4 μg/mL) and hosts of *mcr* genes. Otherwise, the intrinsic colistin resistance was abundant in studied samples compared to the acquired colistin resistance. We reported here colistin resistance genes (*mcr*) in various domestic animals for food consumption in France. Colistin is considered as a last resort antibiotic in France, and resistance to colistin in domestic animals is still prevalent. The factors inducing the dissemination of colistin resistance are multifactorial but are mainly via MGEs. MGEs carrying *mcr* genes easily promote the transmission of these genes from one ecosystem to another. The zoonotic spread of *mcr* genes should be investigated further to reduce the health risks associated with colistin resistance.
